# Supplementary figures and images for: Multi‐Omics Analysis of Human Blood Cells Reveals Unique Features of Age‐Associated Type 2 CD8 Memory T Cells
Source: Aging Cell. 2026 Feb 1;25(2):e70393. doi: 10.1111/acel.70393 (PMC12862018; doi:10.1111/acel.70393)

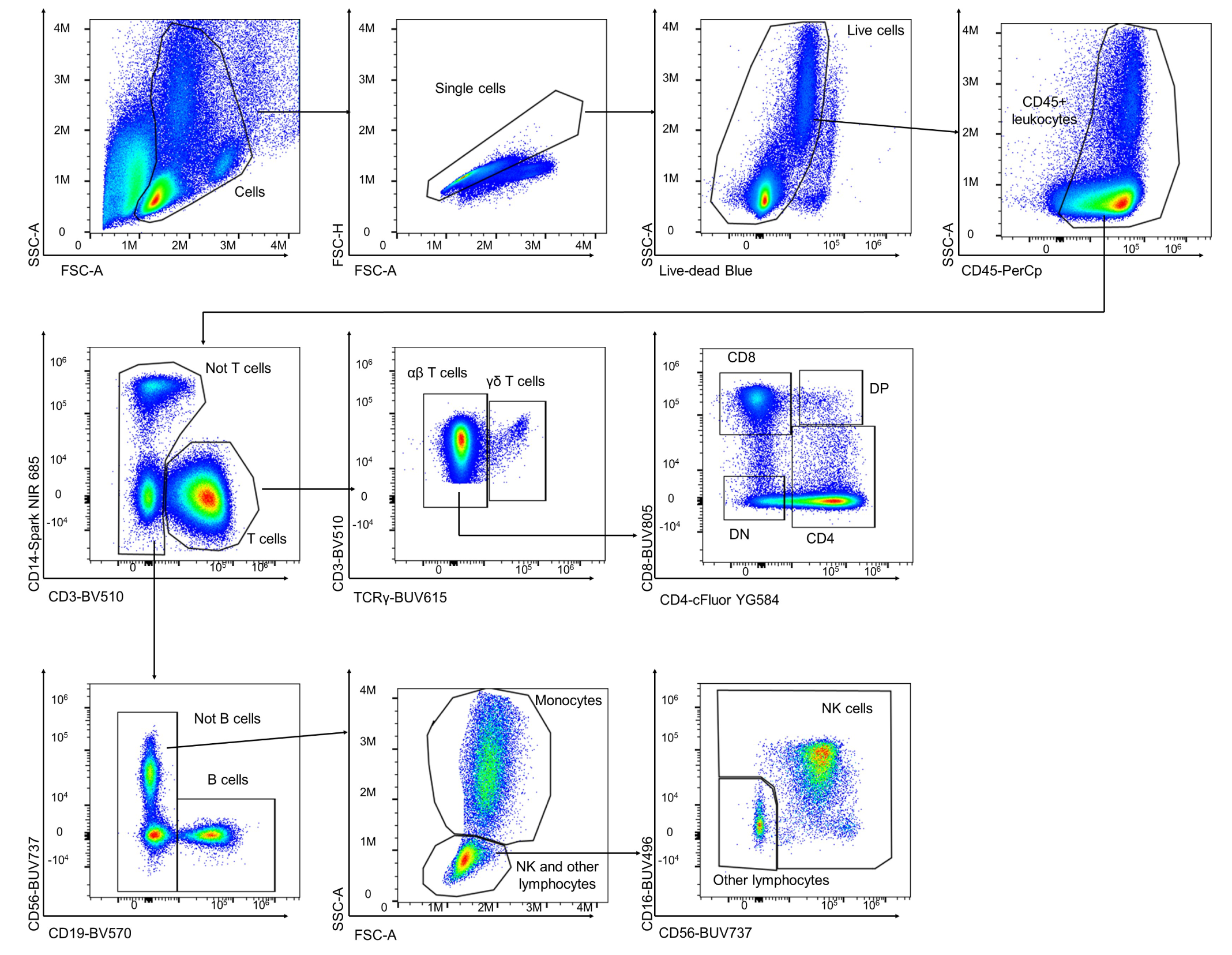

Supplement: Supplementary file 1 — Figure S1: Gating strategy for PBMC subsets with spectral flow cytometry. Gating strategy used for identifying PBMC subsets (B cells, NK. cells, Monocytes, CD4 T cells, CD8 T cells, DN T cells, DP T cells, γδ T cells and other lymphocytes) with spectral flow cytometry are shown. [file ACEL-25-e70393-s007.tif]

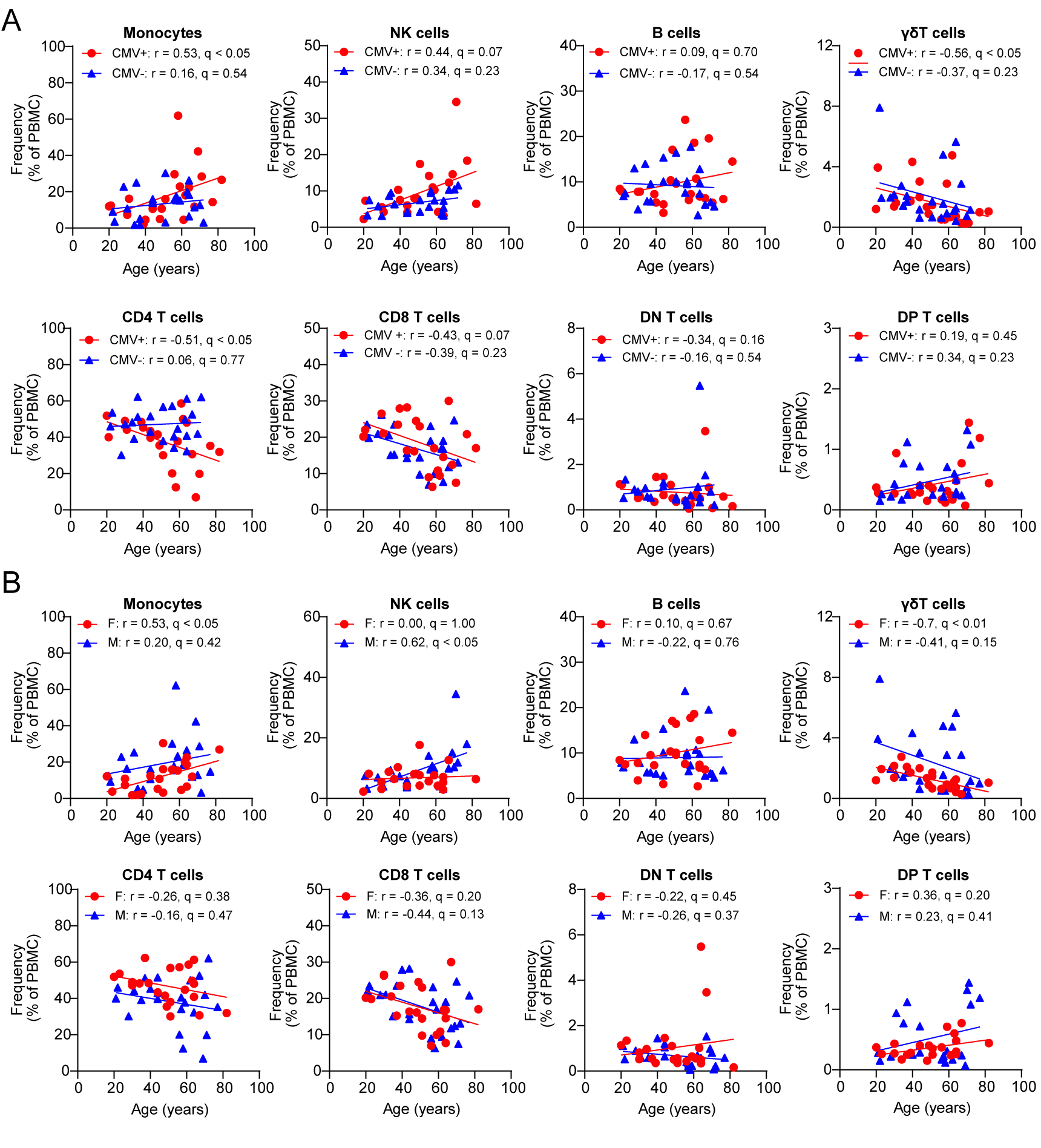

Supplement: Supplementary file 2 — Figure S2: Cell composition changes with age in PBMCs based on donor groups. A. Cell composition changes with age in PBMCs in CMV positive (red) or negative (blue) donors (CMV+ n = 22, CMV‐ n = 23, Spearman correlation test with Benjamini‐Hochberg correction). (B) Cell composition changes with age in PBMCs in female (red) or male (blue) donors (Male n = 23, female n = 22, Spearman correlation test with Benjamini‐Hochberg correction). [file ACEL-25-e70393-s010.tif]

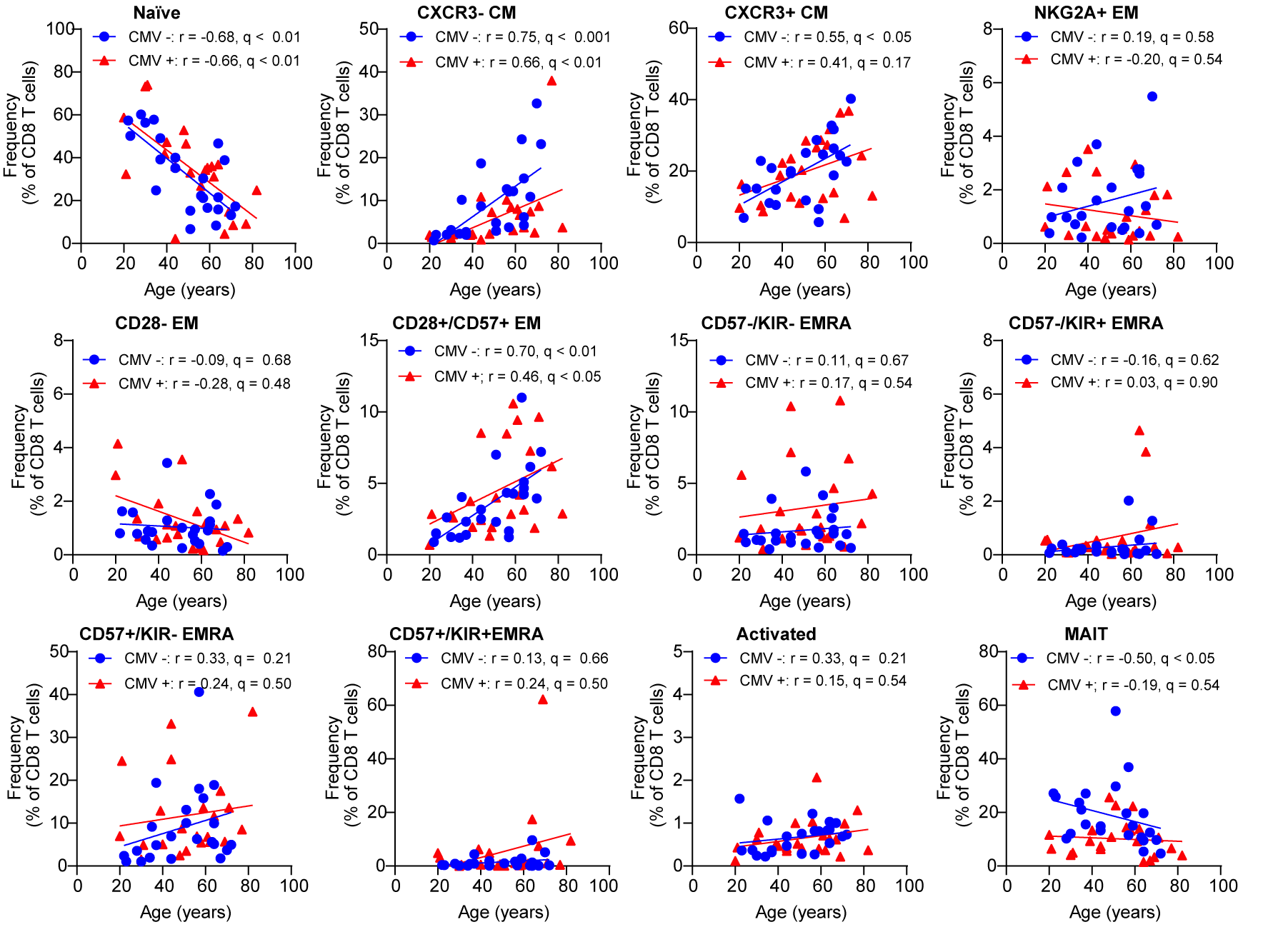

Supplement: Supplementary file 3 — Figure S3: Cell composition changes with age in CD8 T cell subsets based on CMV serostatus. Cell composition changes with age in CD8 T cell subsets in CMV positive (red) or negative (blue) donors are shown (CMV+ n = 22, CMV‐ n = 23, Spearman correlation test with Benjamini‐Hochberg correction). [file ACEL-25-e70393-s005.tif]

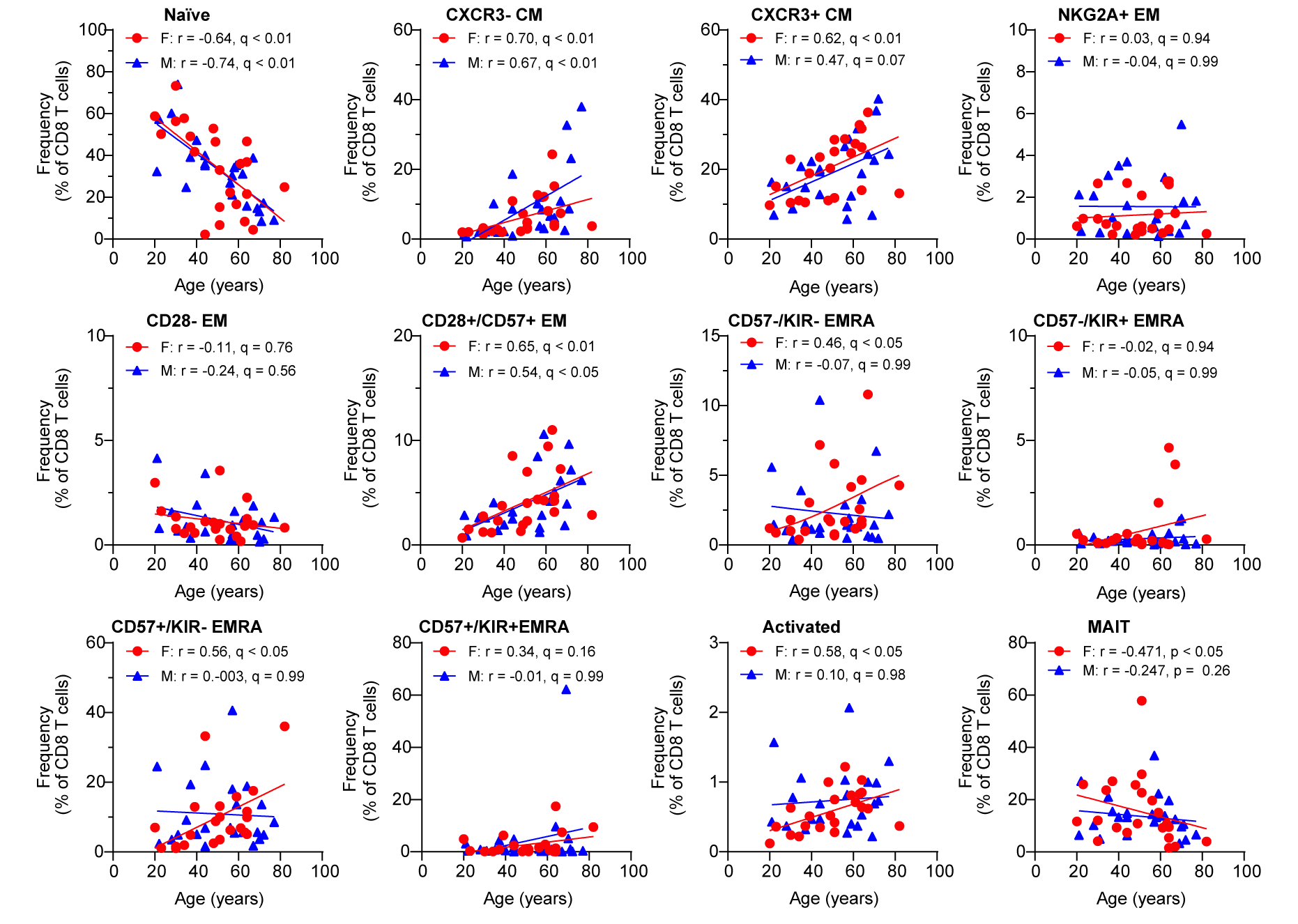

Supplement: Supplementary file 4 — Figure S4: Cell composition changes with age in CD8 T cell subsets based on sex. Cell composition changes with age in CD8 T cell subsets in female (red) or male (blue) donors are shown (female n = 22, male n = 23, Spearman correlation test with Benjamini‐Hochberg correction). [file ACEL-25-e70393-s011.tif]

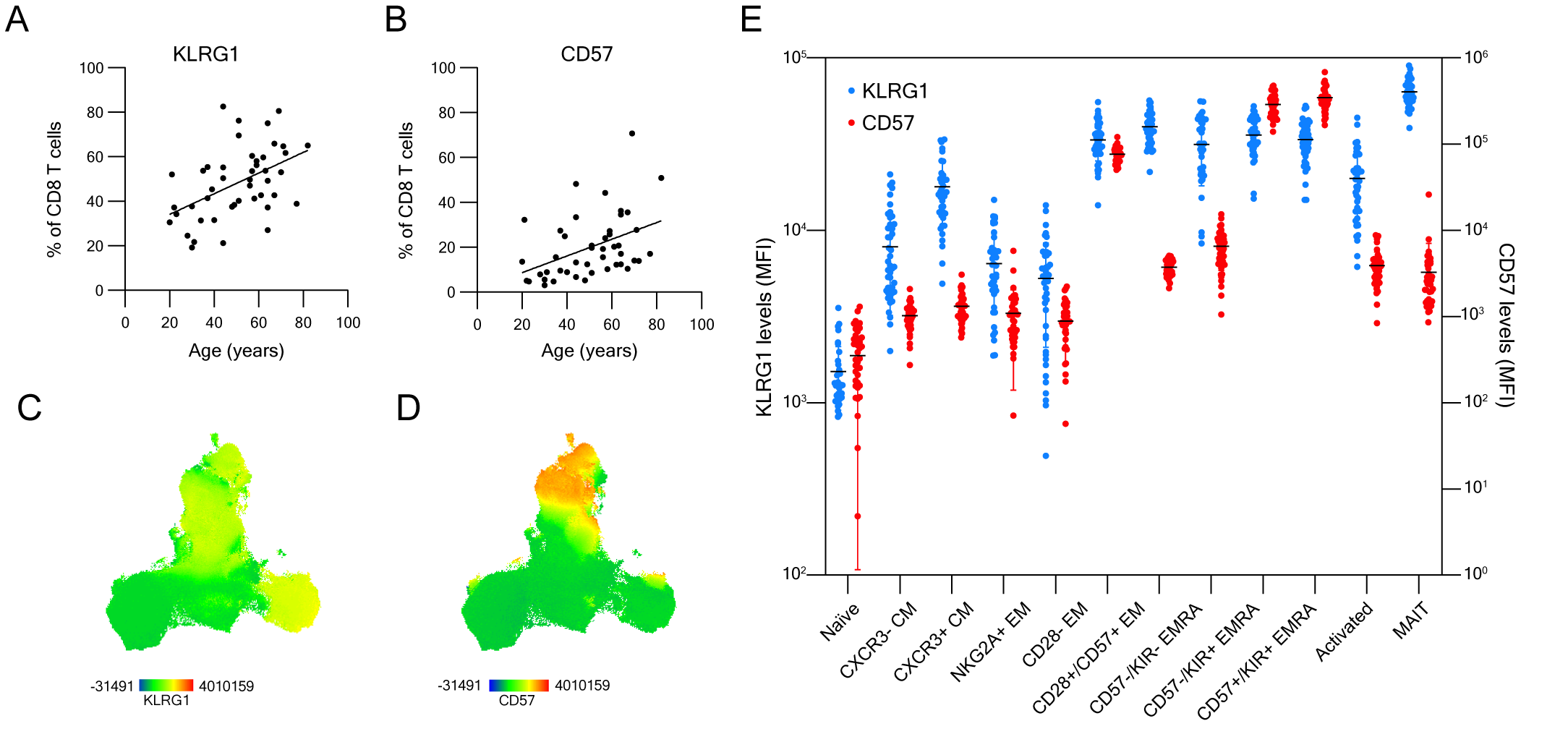

Supplement: Supplementary file 5 — Figure S5: Expression of senescence markers in CD8 T cells. (A) KLRG1 expression changes with age in CD8 T cell (n = 45, Spearman correlation test). (B) CD57 expression changes with age in CD8 T cell (n = 45, Spearman correlation test). (C) UMAP plot with the expression of KLRG1. (D) UMAP plot with the expression of CD57. (E) Expression levels of senescence markers in individual CD8 T cell subsets. KLRG1 (light blue) and CD57 (red) are shown (n = 45). The both left and right y‐axes are shown in a logarithmic scale. [file ACEL-25-e70393-s015.tif]

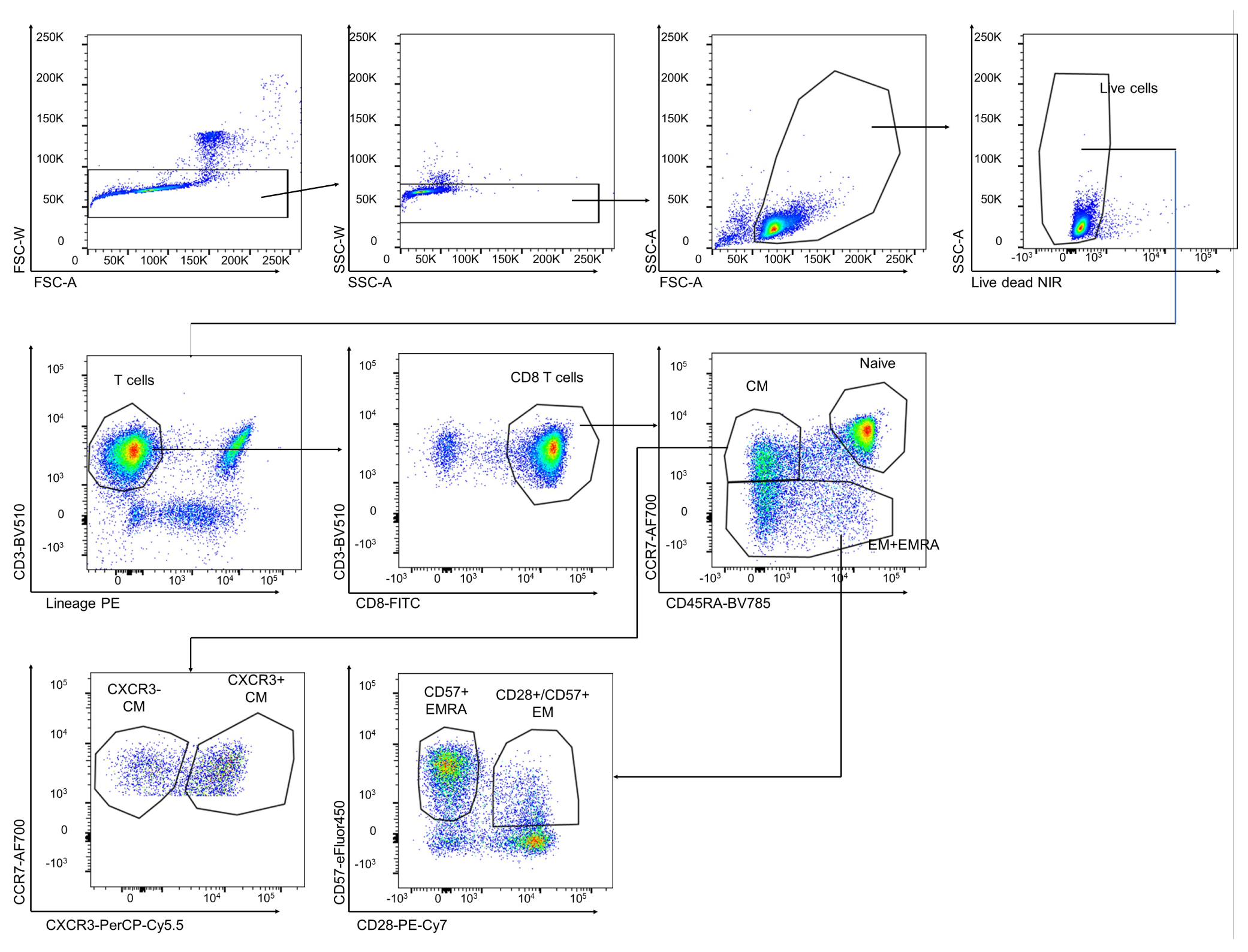

Supplement: Supplementary file 6 — Figure S6: Gating strategy for FACS sorting of CD8 T cell subsets. Gating strategy used for sorting CD8 T cell subsets (Naïve, CXCR3‐ CM, CXCR3+ CM, CD28+/CD57+ EM and CD57+ EMRA) are shown. [file ACEL-25-e70393-s001.tif]

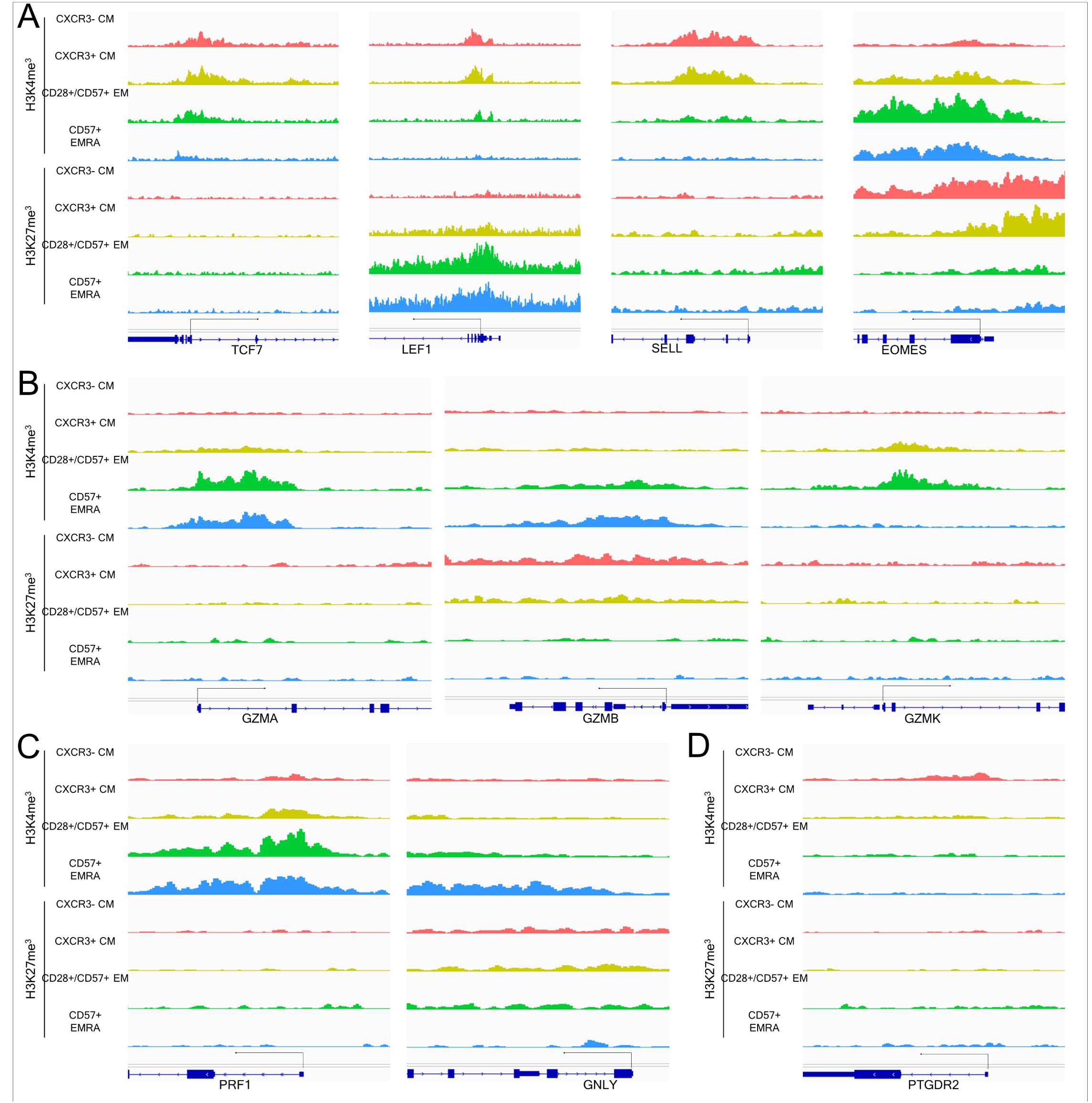

Supplement: Supplementary file 7 — Figure S7: CUT & RUN revealed that each CD8 T cell subset has a distinct pattern of epigenetic features. (A) IGV signal track of CUT & RUN with H3K4me3 and H3K27me3 at T cell transcription factor (TCF7, LEF1, SELL and EOMES) loci. IGV signal track of individual T cell subsets are shown in different colors (CXCR3‐ TCM (red), CXCR3+ TCM (yellow), CD28+/CD57+ EM (green) and CD57+ TEMRA (light blue)). (B) IGV signal track of CUT & RUN with H3K4me3 and H3K27me3 at granzymes (GZMA (left), GZMB (middle) and GZMK (right)) loci. (C) IGV signal track of CUT & RUN with H3K4me3 and H3K27me3 at PRF1 and GNLY loci. (D) IGV signal track of CUT & RUN with H3K4me3 and H3K27me3 at Th2 markers (PTGDR2) loci. [file ACEL-25-e70393-s013.tif]

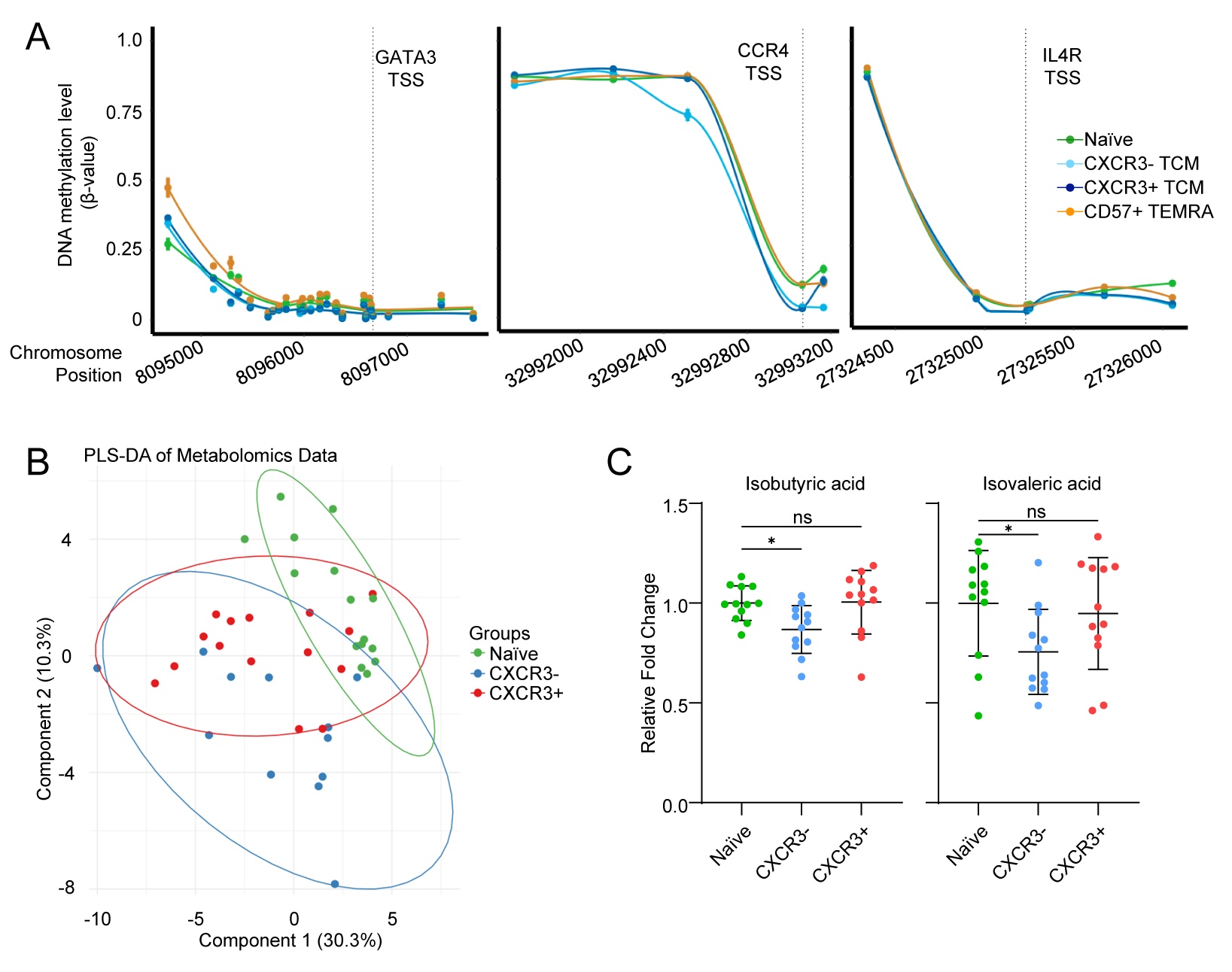

Supplement: Supplementary file 8 — Figure S8: DNA methylation levels at Th2 transcription factor (GATA3) and Th2 markers (CCR4 and IL4R) in each CD8 T cell subset. (A) DNA methylation levels of individual CD8 T cell subsets (Naïve (green), CXCR3‐ TCM (light blue), CXCR3+ TCM (dark blue) and CD57+ TEMRA (orange)) at Th2 transcription factors (GATA3, left) and Th2 markers (CCR4, middle and IL‐4R, right). (B) Partial Least Squares‐Discriminant Analysis (PLS‐DA) plot illustrating distinct separation among the three groups: CXCR3−, CXCR3+, and Naïve. Each group is encircled by a 95% confidence ellipse to indicate the variation within the groups. (C) Bar plots depicting the fold change of isobutyric acid and isovaleric acid in CXCR3‐ and CXCR3+ CD8 T cell subsets relative to naïve CD8 T cells. Data were normalized to CUDA internal standard, and statistical analysis was performed using analysis of variance (ANOVA). Significance levels are denoted as follows: nonsignificant (ns), p > 0.05; *. [file ACEL-25-e70393-s004.tif]

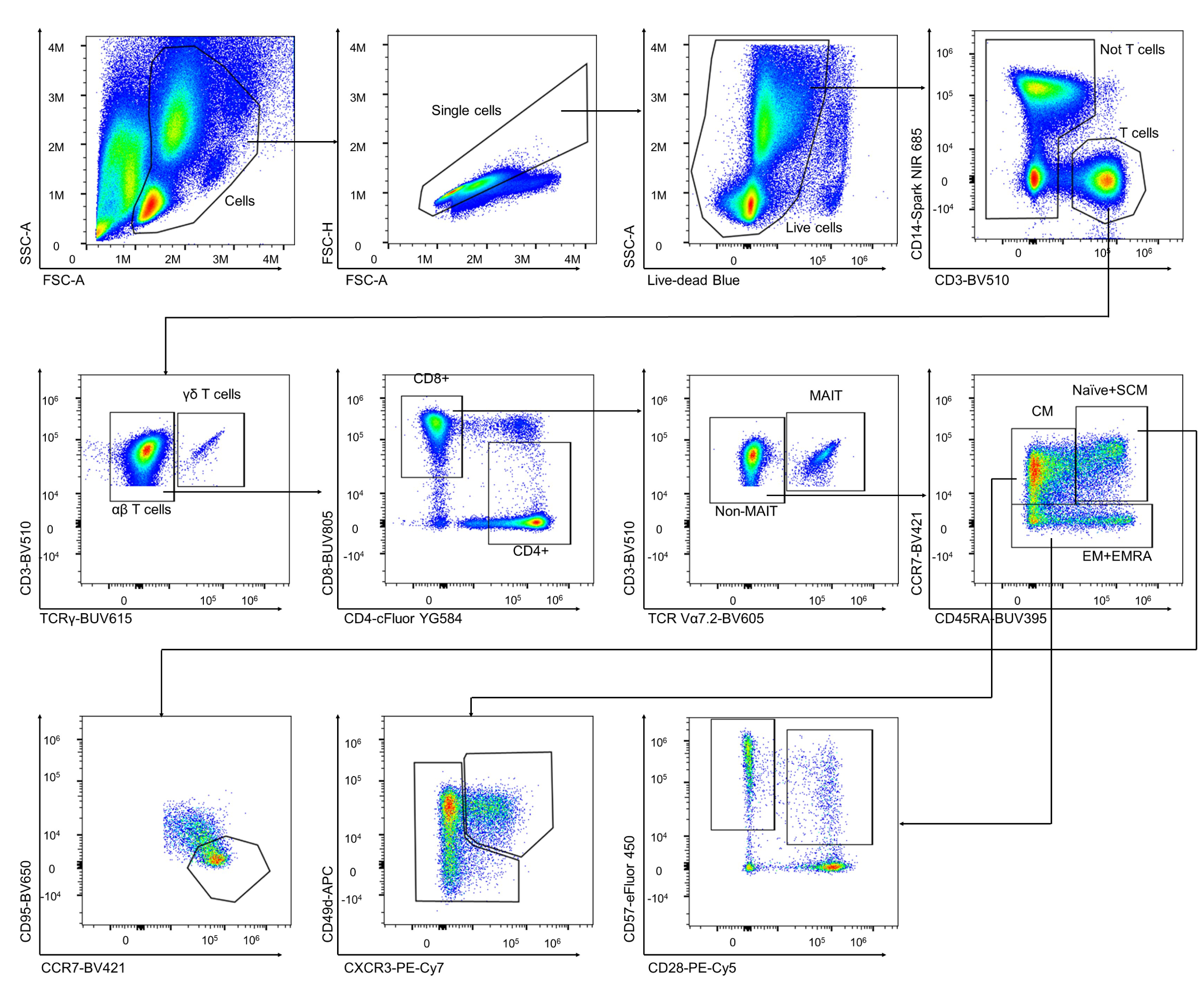

Supplement: Supplementary file 9 — Figure S9: Gating strategy for CD8 T cell subsets with spectral flow cytometry. Gating strategy used for identifying CD8 T cell subsets (Naïve, CXCR3‐ CM, CXCR3+ CM, CD28+/CD57+ EM and CD57+ EMRA) with spectral flow cytometry are shown. [file ACEL-25-e70393-s008.tif]

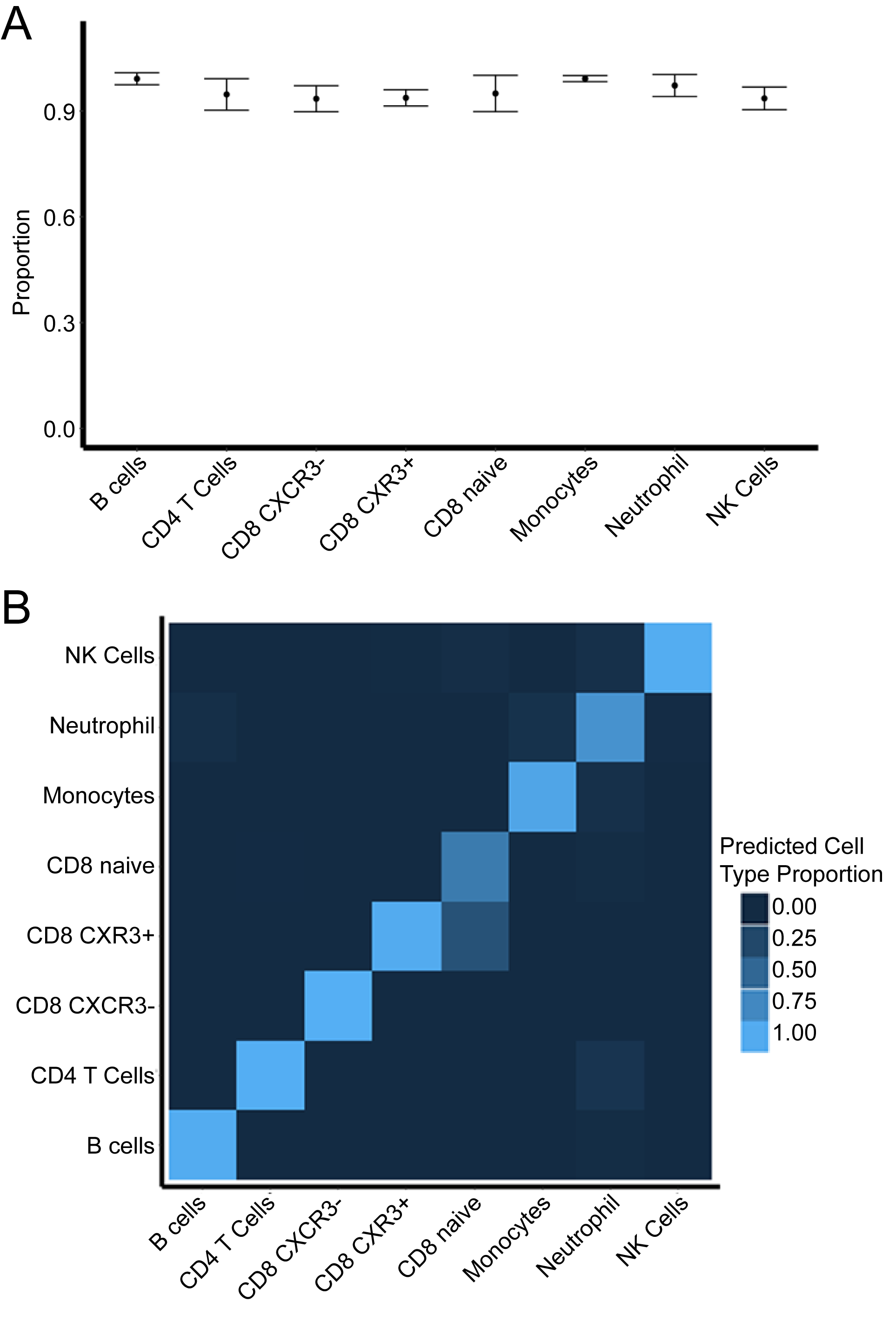

Supplement: Supplementary file 10 — Figure S10: Validation for predictions of cell type proportions. (A) Accuracy of predicting cell type proportion of the corresponding cell type in a sample of 100% sorted cells of one given cell type. Perfect predictions would be reflected by a proportion of 1 on the corresponding cell type. (B) Prediction of proportions of each cell type in a sample of sorted cells of a single given cell type. Perfect predictions would be reflected by a prediction proportion of 1 on the diagonal. [file ACEL-25-e70393-s016.tif]
